# Emotional and behavioural difficulties in gender minority compared to cisgender adolescents: identity specific findings from a contemporary national study

**DOI:** 10.1111/jcpp.70050

**Published:** 2025-09-10

**Authors:** Nicholas Page, Lianna Angel, Sophie Borgia, Colleen Reynolds, Dougie Zubizarreta, Honor Young, Max R. Ashton, James White

**Affiliations:** ^1^ Centre for Development, Evaluation, Complexity and Implementation in Public Health Improvement (DECIPHer), School of Social Sciences Cardiff University Cardiff UK; ^2^ The School Health Research Network (SHRN) Cardiff University Cardiff UK; ^3^ Department of Epidemiology Harvard T.H. Chan School of Public Health Boston MA USA; ^4^ Department of Social and Behavioral Sciences Harvard T.H. Chan School of Public Health Boston MA USA; ^5^ Centre for Trials Research, School of Medicine Cardiff University Cardiff UK

**Keywords:** gender identity, emotional and behavioural difficulties, adolescents, school survey, United Kingdom

## Abstract

**Background:**

Gender minority adolescents are more likely to report emotional and behavioural difficulties compared to their cisgender peers. However, little is known about these experiences for adolescents with specific gender minority identities.

**Methods:**

Cross‐sectional data were obtained from the 2021/22 Student Health and Well‐being survey, a national survey of 11–16‐year‐olds in Wales, UK. Emotional and behavioural difficulties were measured using the Strengths and Difficulties Questionnaire. Gender identity and assigned sex at birth were self‐reported. Multivariable linear regressions with robust standard errors were used to examine associations between gender identity and emotional and behavioural difficulties, adjusting for age, ethnicity, household‐level affluence and correction for multiple testing.

**Results:**

Of the 122,766 participants, 2.0% (2,455) identified as a person with a gender minority identity. Twenty‐eight gender minority identities were self‐reported, with the most prevalent being transgender boy and nonbinary assigned female at birth (both 0.6%). Young people assigned female at birth comprised 80% of gender minority adolescents. In the adjusted model, emotional and behavioural difficulties were reported most frequently by people who identified as non‐binary (*B* = 7.66, 95% CI 7.25, 8.06) and another gender identity (*B* = 7.86, 95% CI 7.34, 8.38), then transgender (*B* = 5.05, 95% CI 4.58, 5.51), when compared to cisgender adolescents. Female sex assigned at birth was associated with more reported difficulties than male sex assigned at birth for adolescents with a transgender or cisgender identity, but not a nonbinary identity.

**Conclusions:**

In this population‐based study, emotional and behavioural difficulties were reported most frequently by adolescents who identified as nonbinary and another gender identity, then transgender, then cisgender. Health and educational practitioners need to be aware that emotional and behavioural difficulties differ across gender minority identities.

## Introduction

Gender minority adolescents (those whose gender identity is different from their assigned sex at birth) have consistently been found to report more symptoms of depression, anxiety and suicide attempts than their cisgender (those whose gender identity is congruent with their assigned sex at birth) peers (Clark et al., 2014; Perez‐Brumer, Day, Russell, & Hatzenbuehler, 2017; White et al., 2023; White, Trinh, & Reynolds, 2023). These studies have, however, tended to be with United States (US) collegiate populations (Lipson, Raifman, Abelson, & Reisner, 2019), involved small samples with reported gender identities collapsed into a single ‘minority’ category that masks effects faced by people with specific identities (Chew et al., 2020; Pinna et al., 2022) and have not recorded assigned sex at birth and gender identity (White, Moore, et al., 2023), preventing examination of their intersection (Guz et al., 2021; Perez‐Brumer et al., 2017).

In western societies, today's generation of adolescents arguably live in a more socially progressive environment. The United Kingdom's (UK) 2010 Equality Act offers legal protection against discrimination according to race, sexual identity and for people undergoing gender reassignment (Equality Act, 2010). However, in recent years, the advent of gender politics has shifted the cultural narrative such that the legitimacy and rights of gender minority people are often in debate. In England and Wales in 2024, transgender hate crimes accounted for 3% of hate crimes reported, up from 1% in 2014; in comparison, race hate crimes have reduced by 10% since their peak in 2022 (United Kingdom Government, 2024). Furthermore, gender minority adolescents consistently report experiencing more bullying than their cisgender peers (Johns et al., 2019; White, Trinh, & Reynolds, 2023). Given the shifting social climate in the United Kingdom, there is a need to examine the emotional and behavioural difficulties reported by the current generation of adolescents by their gender identity.

In the few studies which examine reported symptoms by reported gender identity and assigned sex at birth, one US collegiate sample (*n* = 65,213, ≥18 years of age) found that while all gender minority identities reported more probable depressive, anxiety, eating disorders and suicidal plans and attempts, transmasculine students (gender minority students assigned female at birth [AFAB]) and genderqueer students were particularly vulnerable (Lipson et al., 2019). Another small study (*n* = 241) using convenience sampling in 16‐ to 25‐year‐olds from the United Kingdom found gender minority participants who were AFAB reported more symptoms of depression and anxiety than their gender minority peers who were AMAB or cisgender (Hunter, Butler, & Cooper, 2021).

To address these research gaps, we examined emotional and behavioural difficulties reported in a large nationally representative random sample of more than 120,000 11‐to‐16‐year‐olds in Wales, UK. As far as the authors are aware, this is the first study in the United Kingdom to examine emotional and behavioural difficulties in high school‐aged adolescents by gender identity and assigned sex at birth.

## Methods

### Study design and ethics

The School Health Research Network (SHRN) Student Health and Well‐being (SHW) survey is a school‐based cross‐sectional survey in Wales, UK (www.shrn.org.uk) (Page et al., 2024). All state secondary schools in Wales (*n* = 205) and a small number of independent (‘private’) schools (*n* = 7) are eligible for inclusion. We used data gathered in the 2021/22 survey from September 2021 to January 2022. The anonymous survey was completed online by students during school hours (Page et al., 2024).

Ethical approval for the 2021/22 survey was obtained from Cardiff University School of Social Sciences Research Ethics Committee (SREC/4251). Consent to participate in the survey was required at three levels: school, parent/carer and student. School‐level consent was obtained at survey registration. Parents/carers were informed about the survey through at least two methods of communication (i.e., letter, email, text message or web‐based app) followed by a process of opt‐out consent. Student assent was obtained at the beginning of the survey. Students were informed that their participation was voluntary, that they could choose not to answer any question (except for year group and consent) by selecting, ‘I do not want to answer’, and that they could withdraw from the survey at any point. This manuscript adheres to the STROBE reporting guidelines (von Elm et al., 2007).

### Measures

Gender identity and assigned sex at birth were self‐reported. Assigned sex at birth was reported with the question, ‘Were you described as male or female at birth?’, with the response options of: ‘male’, ‘female’ and ‘I do not want to answer’. Gender identity was reported using the question ‘Are you a boy or a girl?’ with the response options of ‘boy’, ‘girl’, ‘neither word describes me’ and ‘I do not want to answer’. Students who selected ‘neither word describes me’ were able to write their identity in an open response box with the prefix, ‘I identify myself as…’. We used these responses to derive five gender identities: cisgender, transgender, nonbinary, another gender identity and multiple identities. Categorisation as transgender was determined using responses to the gender and assigned sex at birth questions (e.g., boy, female) or via self‐identification through the open response option. For all other gender minority identities, students had to write the identity to be classified in this way. If participants wrote two identities that could have been interpreted as one being within another (e.g., nonbinary, genderfae) they were categorised as having multiple identities. Students who selected ‘neither word describes me’ but did not complete the open response were defined as unspecified as we could not confirm their gender identity. We have not categorised these people as a gender minority but report them here as this pattern of nonresponse may be informative.

Next, we examined both assigned sex at birth and gender identity to create 12 groups: cisgender boy (boy, assigned male at birth [AMAB]), cisgender girl (girl, assigned female at birth [AFAB]), transgender boy (boy, AFAB), transgender girl (girl, AMAB), nonbinary AMAB, nonbinary AFAB, another gender identity AMAB, another gender identity AFAB, unspecified gender identity AMAB, unspecified gender identity AFAB, multiple gender identities AMAB and multiple gender identities AFAB.

Emotional and behavioural difficulties were measured using the adolescent self‐completed Strengths and Difficulties Questionnaire (SDQ) (Goodman & Goodman, 2009). The SDQ is a validated screening tool to measure child and adolescent behavioral and emotional difficulties. The SDQ assesses conduct problems, hyperactivity, emotional symptoms, peer relationship problems and prosocial behaviour, experienced over the last 6 months. Following the published method (Goodman & Goodman, 2009), individual item scores were summed to create subscale scores ranging from 0 to 10. The total score included four of the five subscales (exc. prosocial behaviour) and ranged between 0 and 40.

Demographic characteristics included age, household‐level affluence measured using the Family Affluence Scale (FAS) (Hartley, Levin, & Currie, 2016) and ethnicity (i.e., White: ‘White British’, ‘White Irish’, ‘White Gypsy or Irish traveller’, ‘White Roma’, ‘White Other’; ethnic minority: ‘African’, ‘Caribbean or Black’, ‘Pakistani’, ‘Indian’, ‘Bangladeshi’, ‘Chinese’, ‘Mixed or Multiple’, ‘Arab’, ‘Other’). FAS is a composite measure which includes six items capturing car, computer and dishwasher ownership, bedroom occupancy, number of household bathrooms and frequency of family holidays. Individual items were summed to form a scale score ranging from 0 to 13. Scores were categorised into low, medium and high affluence tertiles for analyses.

Other measures included weekly smoking status (‘at least once a week’), cannabis use in the last 30 days, screening for adolescent alcohol use disorder (drinking two or more alcoholic drinks per drinking episode) (Clark et al., 2016), bullying victimisation at school in the past couple of months and the Short Warwick‐Edinburgh Mental Well‐being Scale (SWEMWBS; Tennant et al., 2007).

### Statistical analysis

To describe the characteristics of adolescents according to gender identity, we used univariable linear regression with robust standard errors (to account for students being nested within schools). Three separate multivariable models were then performed for the association between gender identity and emotional and behavioural difficulties to examine the potential confounding effects of (1) age, (2) ethnicity and (3) age, ethnicity and household‐level affluence. Models were then estimated again to examine effects at the intersection of gender identity and assigned sex at birth. Results are presented as regression coefficients with 95% confidence intervals (CIs). A sensitivity analysis was conducted after excluding participants with any missing data. Analyses were performed in Stata MP version 14.2 (Stata Corp).

Inappropriate responses on gender were omitted prior to imputation: these included disingenuous responses (e.g., ‘I am cool’, ‘lizard’, ‘God’) and probable misunderstandings (e.g., sexualities, nationalities) (*n* = 271). Participants whose age did not correspond with their reported UK school year were also omitted (*n* = 55). Adolescents who reported multiple gender minority identities (*n* = 69) or who were categorised as another gender minority AMAB (*n* = 43) were excluded due to smaller numbers.

Missing data per variable ranged from 0% to 15.3%. Missing data in all variables (exposures, outcomes and covariates) were addressed through multiple imputation using chained equations. We assumed data were missing at random. We used a multivariate normal imputation model to impute all missing data. Each model included all variables, including the following auxiliary variables: an indicator variable for each school, weekly smoking status, cannabis use in the last 30 days, screening for adolescent alcohol use disorder, bullying victimisation and SWEMWBS. Estimates were obtained by pooling results across 40 imputed data sets using the Rubin rules, and assessment of Monte Carlo errors suggested that this was a suitable number of imputations (White, Royston, & Wood, 2011). To reduce the risk of generating spurious findings due to multiple testing, the threshold for significance was Bonferroni adjusted to *p* < .004 (*p* = .05/12).

## Results

From the 123,204 11–16‐year‐olds who participated, 122,766 remained after data cleaning and made up the imputed analytical sample. Of these, 2.0% (2,455) were coded as a gender minority (full breakdown listed in Table S1). Among gender minority adolescents, 45% (*n* = 1,104) identified as transgender, 35% (*n* = 860) nonbinary and 20% (*n* = 491) another gender identity. Participants who were AFAB made up 80% of gender minority adolescents: 30% (*n* = 737) identified as transgender boy, 30% (*n* = 737) as nonbinary AFAB and 20% (*n* = 491) another gender identity AFAB. Among gender minority adolescents AMAB, 15% (*n* = 367) identified as transgender girl and 5% (*n* = 123) as nonbinary AMAB. There were 1,105 adolescents AFAB and 246 AMAB with an unspecified gender identity. Details of the 24 unique gender identities included in the ‘another’ gender identity category are provided in Table S2.

Table 1 shows the characteristics of adolescents by their reported gender identity and assigned sex at birth. Mean age was higher among adolescents with unspecified gender AMAB and non‐binary adolescents AFAB but lower among those with unspecified gender AFAB, compared to cisgender boys (all *p* < .05). A higher percentage of transgender girls (16.8%, *p* = .017) and those with unspecified gender AMAB (21.0%, *p* < .001) and a lower percentage of cisgender girls (10.8%, *p* = .002) reported ethnic minority identities relative to cisgender boys (11.6%). Household‐level affluence was highest among cisgender boys (24.8%) with comparatively lower percentages of nonbinary adolescents AMAB (11.9%, *p* < .001), transgender boys (13.2%, *p* < .001) and nonbinary adolescents AFAB (11.2%, *p* < .001) living within high socioeconomic status households. Identities other than cisgender boys were more likely to report in‐person bullying, with victimisation highest among nonbinary adolescents (AMAB: 70.8%, *p* < .001; AFAB: 67.1%, *p* < .001). Alcohol, tobacco and cannabis use were more common in gender minority than cisgender participants, with use highest among transgender girls (all *p*'s < .05).

**Table 1:** Characteristics according to gender identity

	Cisgender (*n* = 118,960)		Gender minority (*n* = 2,455)					Unspecified (*n* = 1,351)	
	Boy (*n* = 60,524)^a^	Girl (*n* = 58,436)^a^	Boy/transboy (*n* = 737)^b^	Girl/transgirl (*n* = 367)^b^	Nonbinary AMAB (*n* = 123)^c^	Nonbinary AFAB (*n* = 737)^c^	Another gender identity AFAB (*n* = 491)^d^	Unspecified AMAB (*n* = 246)^e^	Unspecified AFAB (*n* = 1,105)^e^
Age, mean (*SD*)	13.2 (0.2)	13.2 (0.2)	13.2 (0.2)	13.2 (0.3)	13.3 (0.2)	13.4 (0.2)	13.4 (0.2)	13.6 (0.2)	13.0 (0.2)
FAS, % (95% CI)									
Low (0–8)	43.4 (43.4, 43.5)	44.8 (44.7, 44.8)	60.7 (60.1, 61.2)	51.5 (50.7, 52.4)	62.0 (60.7, 63.2)	60.1 (59.6, 60.6)	57.1 (56.4, 57.8)	54.6 (53.7, 55.5)	58.9 (58.4, 59.3)
Medium (9–10)	31.8 (31.7, 31.8)	30.8 (30.7, 30.9)	26.1 (25.6, 26.6)	25.3 (24.5, 26.0)	26.1 (25.0, 27.2)	25.6 (25.1, 26.1)	30.9 (30.3, 31.6)	29.0 (28.2, 29.9)	28.2 (27.8, 28.6)
High (11–13)	24.8 (24.8, 24.9)	24.4 (24.4, 24.5)	13.2 (12.9, 13.6)	23.2 (22.5, 23.9)	11.9 (11.1, 12.7)	14.3 (13.9, 14.7)	12.0 (11.5, 12.4)	16.4 (15.7, 17.1)	12.9 (12.6, 13.2)
Ethnic minority, % (95% CI)	11.6 (11.5, 11.6)	10.8 (10.8, 10.9)	12.7 (12.3, 13.1)	16.8 (16.2, 17.5)	13.6 (12.7, 14.4)	11.2 (10.8, 11.5)	10.9 (10.5, 11.4)	21.0 (20.3, 21.8)	11.6 (11.3, 11.9)
Bullied in past few months, % (95% CI)	28.8 (28.8, 28.9)	35.0 (34.9, 35.1)	58.3 (57.8, 58.9)	51.6 (50.7, 52.5)	78.0 (77.0, 79.1)	67.7 (67.2, 68.2)	64.6 (63.9, 65.3)	52.0 (51.0, 52.9)	57.9 (57.5, 58.4)
≥2 alcoholic drinks per drinking episode, % (95% CI)	18.3 (18.2, 18.3)	22.9 (22.8, 23.0)	24.2 (23.7, 24.7)	26.0 (25.2, 26.8)	22.2 (21.2, 23.3)	22.3 (21.8, 22.7)	19.1 (18.6, 19.7)	32.5 (31.6, 33.3)	18.5 (18.1, 18.9)
Weekly smoker, % (95% CI)	2.9 (2.9, 2.9)	3.3 (3.3, 3.3)	7.5 (7.3, 7.8)	10.7 (10.2, 11.2)	3.8 (3.3, 4.3)	5.9 (5.6, 6.1)	1.0 (0.9, 1.1)	15.5 (14.9, 16.2)	3.7 (3.6, 3.9)
Used cannabis in past 30 days, % (95% CI)	3.8 (3.8, 3.9)	4.1 (4.0, 4.1)	7.0 (6.7, 7.3)	10.2 (9.7, 10.7)	6.7 (6.0, 7.3)	4.8 (4.6, 5.1)	1.9 (1.7, 2.1)	15.4 (14.7, 16.0)	3.3 (3.1, 3.4)
Mental well‐being, mean (*SD*)^f^	24.4 (0.8)	21.9 (0.8)	18.3 (0.9)	20.2 (1.1)	18.9 (0.8)	17.3 (0.7)	17.5 (0.8)	19.5 (1.0)	17.6 (0.8)

^a^ Gender aligns with assigned sex at birth.

^b^ Gender contrasts with assigned sex at birth.

^c^ Self‐identified as nonbinary gender (assigned male at birth, AMAB/assigned female at birth, AFAB).

^d^ Self‐identified as gender minority identity other than nonbinary. Reported identities include agender, bigender, demi‐girl, demi‐boy, genderfluid, genderqueer, transmasculine, pangender, boyflux, demigender, femboy, genderdoe, genderfae, genderfaun, genderflux, gendergender, gendervoid, intergender, librafemme, librafluid, omnigender, paragirl, polygender and tomboy.

^e^ Self‐identified gender as ‘neither word describes me’ but provided no additional information.

^f^ Short Warwick‐Edinburgh Mental Well‐being Scale.

**Figure 1:**
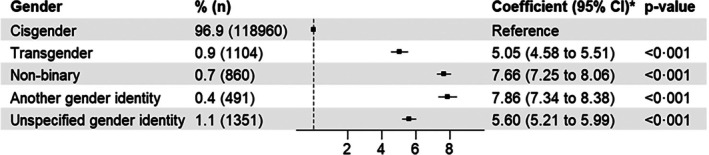
Associations between gender identity and emotional and behavioral difficulties. *Regression coefficient adjusted for age, ethnicity, household‐level affluence

Gender minority adolescents reported more emotional and behavioural difficulties than their cisgender peers. Figure 1 shows that after adjusting for age, ethnicity and household‐level affluence and correction for multiple testing, emotional and behavioural difficulties were most frequently reported by adolescents who were non‐binary and another gender identity, then transgender, then cisgender.

**Figure 2:**
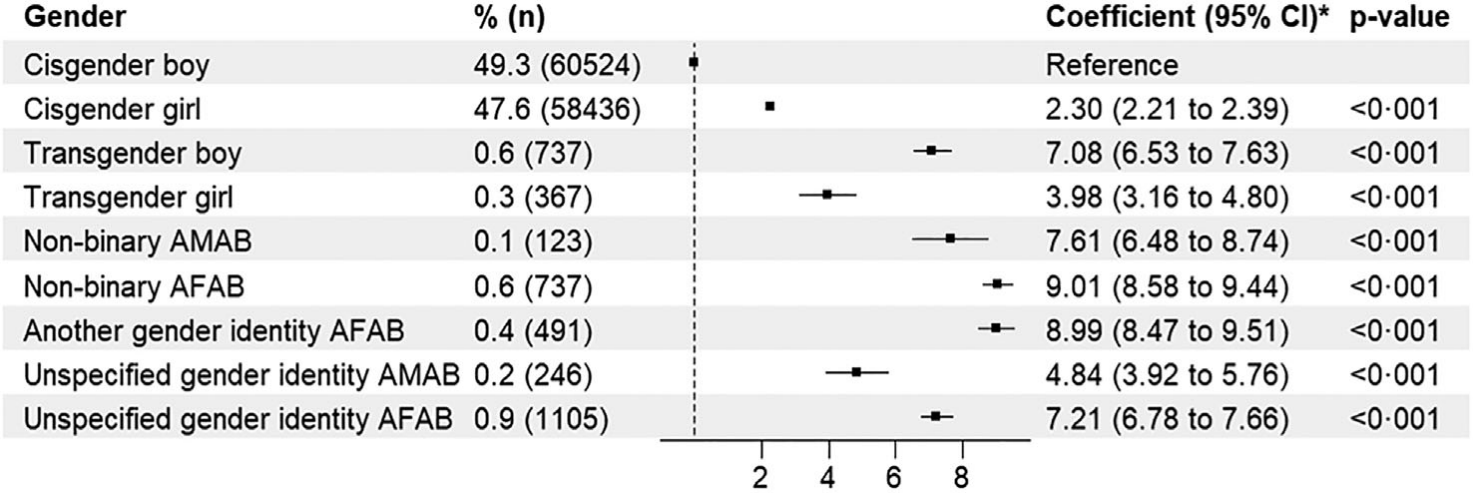
Associations between gender identity and assigned sex at birth with emotional and behavioural difficulties. AFAB, assigned female at birth; AMAB, assigned male at birth. *Regression coefficient adjusted for age, ethnicity, household‐level affluence

Figure 2 shows that after stratifying gender identity by assigned sex at birth, cisgender and transgender adolescents who were assigned female at birth reported more difficulties than those assigned male at birth, but there were fewer differences for nonbinary adolescents. Estimates for adolescents with an unspecified gender identity were more similar in magnitude to those observed for gender minorities than to cisgender identities. Tables S3 and S4 show that adjustment for age, ethnicity and household‐level affluence had little effect on estimates.

Sensitivity analyses shown in Tables S5 and S6 where there was no missing data showed estimates were comparable to those obtained from the imputed data.

## Discussion

The current study used a contemporary population‐based sample to investigate adolescent emotional and behavioural difficulties by gender identity and assigned sex at birth. We found gender minority adolescents reported significantly higher emotional and behavioural difficulties compared with their cisgender peers. These difficulties were most frequently reported by nonbinary adolescents and adolescents with another gender minority identity, then transgender, then cisgender adolescents. Female sex assigned at birth was associated with more emotional and behavioural symptoms than male sex assigned at birth for adolescents with a transgender or cisgender identity, but not a nonbinary identity.

### Comparison with existing literature

The prevalence of adolescents in our national sample who were identified as a person with a gender minority identity was small (2.0%) but comparable with estimates from older community samples in North America (2.1%, *n* = 65, 231, Lipson et al., 2019; 1.9%, *n* = 908, Almeida, Johnson, Corliss, Molnar, & Azrael, 2009), as well as nationally representative samples of students in New Zealand (1.2% transgender, *n* = 8,166, Clark et al., 2014), 13‐ and 15‐year‐olds in Scotland (3% transgender or non‐binary, *n* = 2,910, Inchley, Mabelis, Brown, Willis, & Currie, 2023) and a UK sample of 17‐year‐olds (1.5% genderqueer, *n* = 6,022, Ricardo et al., 2024). Here we present disaggregated estimates across multiple gender minority identities. The most common gender minority identities were non‐binary AFAB (30%), transgender boy AFAB (30%), followed by another gender identity AFAB (20%), transgender girl AMAB (15%) and nonbinary AMAB (5%), such that people who were AFAB accounted for 80% of gender minority adolescents. Our findings replicate those of one systematic review which found that across 17 countries those under 18 years of age who were referred to specialist gender clinics were more likely to be AFAB than AMAB (Taylor, Hall, Langton, Fraser, & Hewitt, 2024).

In line with results from studies that sampled adolescents from the general population, we replicated the well‐documented association between gender minority identity and emotional and behavioural difficulties (Guz et al., 2021; White, Trinh, & Reynolds, 2023). Our results extend previous findings by showing that for gender minority adolescents, these difficulties were most elevated in those who identified as non‐binary, followed by transgender adolescents. In the US Healthy Minds Study of transgender and genderqueer US college students (*n* = 65,213), genderqueer AFAB were more likely to report symptoms consistent with probable depression and anxiety than genderqueer AMAB, but no differences were found between transgender AFAB and AMAB (Lipson et al., 2019). Another small study of people attending a national transgender health service in the UK, comparing nonbinary and binary transgender 16–25 year ‐olds (nonbinary = 57, binary = 331), found that nonbinary people reported more symptoms of anxiety and depression than binary transgender young people (Thorne et al., 2019).

Our results are consistent with the existing literature showing that symptoms and diagnoses of depression (Salk, Hyde, & Abramson, 2017) and anxiety (Pigott, 1999) have a higher prevalence in people who report being AFAB than AMAB. In one small UK‐based online convenience sample of 16–25‐year‐old LGBTQ people (*n* = 677), nonbinary or transgender people who were AFAB were more likely to report having a current mental health condition, having self‐harmed and experiencing childhood sexual abuse than people who were nonbinary or transgender and AMAB (Rimes, Goodship, Ussher, Baker, & West, 2017). In our study, across adolescents who reported being cisgender or transgender, those who were AFAB reported more emotional and behavioural problems than their AMAB peers. There were smaller differences between nonbinary adolescents who were AMAB and AFAB. Our findings are consistent with literature that the intersectional effects of being AFAB and a gender minority put transgender AFAB adolescents at a higher risk (Crenshaw, 1991). The reasons these effects were not replicated in nonbinary adolescents are unclear but could be due to smaller numbers identifying as nonbinary AMAB (*n* = 123) in our sample compared to AFAB (*n* = 737), limiting statistical power.

It is well evidenced that adolescent females have worse mental health outcomes on average compared to males (Campbell, Bann, & Patalay, 2021). Potential explanations for this may include differential exposure to certain genetic, cognitive and environmental risk factors (Martin & Hadwin, 2022). For example, adolescent females tend to exhibit higher levels of rumination than males, and this has been shown to mediate the effects of sex on depression (Jose & Brown, 2008). Higher rates of gender inequality in adolescent mental health have also been shown in more gender‐equal countries and countries with higher GDP (Campbell et al., 2021; de Looze et al., 2025). Intersectionality theory emphasises how multiple components of an individual's identity – in this case sex and gender – can interact synergistically to create unique vulnerabilities (Crenshaw, 1991). That adolescents who were AFAB comprised 80% of gender minority youth in this study, and across most identities reported more emotional and behavioural difficulties, highlights the importance of support for adolescents who are gender minorities as well as those who were AFAB.

### Strengths and limitations

A key strength of this study is its use of a large, nationally representative sample, enhancing generalisability to the broader population. Use of a contemporary cohort also provided much needed insight into gender minority mental health given these adolescents have grown up in an era of both sociopolitical changes towards equality and diversity, but also intense public debate on the legitimacy and rights of gender minority people. The detailed categorisation of gender identities and utilisation of a validated outcome enabled a more nuanced exploration of emotional and behavioural symptoms across specific gender identities. However, several limitations warrant consideration. First, the reliance on self‐reported data might introduce reporting bias. Second, we classified people according to the descriptions they provided, which may have resulted in misclassification. Third, small sample sizes for certain gender identity categories reduced statistical power for some comparisons. Some adolescents responded that ‘neither word describes me’ in relation to their gender identity but did not provide further self‐report detail when asked to describe their gender. Further research is needed to understand why some adolescents answer in this way, and then leave more detailed, related questions blank. Fourth, we did not have information on the sexual identity of adolescents. As gender identity and sexual identity are linked, the associations we report on gender identity may be confounded by shared experiences of marginalisation due to sexual and gender minority identity. Finally, the cross‐sectional design precludes causal inference regarding the observed associations between gender identity and mental health outcomes.

### Implications for policy and practice

The elevated emotional and behavioural difficulties reported by gender minority adolescents, particularly nonbinary and transgender individuals, underscores the need for mental health interventions for these groups. Chronic exposure to unique stressors such as discrimination and stigma associated with gender minority status has been posited as an explanation for poorer mental health outcomes among this population (Delozier, Kamody, Rodgers, & Chen, 2020; Valentine & Shipherd, 2018). School environments are modifiable and could help to mitigate exposure to social stressors shown to adversely impact youth mental health, such as peer victimisation, to which gender minorities are particularly vulnerable (Collier, van Beusekom, Bos, & Sandfort, 2013). Examples include Gay‐Straight Alliances, where lesbian, gay, bisexual, transgender and queer youth and their allies attempt to improve the school climate for sexual and gender minority youth (Marx & Kettrey, 2016) and staff education to foster greater acceptance of gender minority identities (Swanson & Gettinger, 2016; Schlief et al., 2023). Systematic review evidence suggests school connectedness is protective against youth anxiety and depression (Raniti, Rakesh, Patton, & Sawyer, 2022). Findings from a small convenience sample of transgender and nonbinary youth in the United States (*n* = 252; mean age, 16.7 years) observed similar protective associations from school connectedness (Parodi et al., 2022), suggesting interventions which increase connectedness in educational settings should be expanded and evaluated.

Nonbinary adolescents reported more emotional and behavioural difficulties than both transgender and cisgender youth. While reasons for this are unclear, previous studies examining health inequalities by sexual identity show individuals identifying as bisexual commonly report poorer outcomes compared to their homosexual peers (e.g., Feinstein & Dyar, 2017; Költő et al., 2020). An explanation posited for this is that bisexuals experience stigma and discrimination owing to perceptions among heterosexual and homosexual populations that bisexuality is not a legitimate identity (Feinstein & Dyar, 2017). Nonbinary status challenges the widely held notion of a gender being binary more so than trans identities, meaning it is possible that nonbinary youth may similarly experience stressors unique to their identity (including nonaffirmation of their gender identity and barriers to accessing appropriate support services: Chew et al., 2020). Effective mental health support for non‐binary youth will likely require clinical approaches that recognise nonbinary identities as distinct from trans identities (Matsuno, 2019).

## Conclusion

In a large nationally representative sample, emotional and behavioural difficulties were most frequently reported by nonbinary and another gender identity adolescents, then transgender, then cisgender adolescents. Female sex assigned at birth was associated with more reported difficulties in cisgender and transgender adolescents than male sex assigned at birth. Our findings are relevant for clinicians and those working in public mental health to be aware that gender minority adolescents and those who are assigned female at birth are at an elevated risk of emotional and behavioural difficulties. Given that these symptoms are often comorbid and exacerbate over time, these adolescents are likely to carry such adverse outcomes into adulthood, with associated social, health and economic costs. Addressing these inequalities should be a priority.

## Ethical considerations

Ethical approval for this study was not required as it involved the analysis of anonymised secondary data. The 2021/22 SHW survey was approved by Cardiff University School of Social Sciences Research Ethics Committee on June 17, 2021 (Ref. SREC/4251). Consent to participate in the survey was required at three levels: school, parent/carer and student.

**Key points**

**What's known?**

Studies suggest gender minority adolescents report more emotional and behavioural difficulties than cisgender individuals, although there is little evidence of experiences among specific minority identities.

**What's new?**

In a contemporary, population‐based study of high school‐aged pupils, we found that emotional and behavioural difficulties were highest among those identifying as either nonbinary or another gender identity, then transgender, then cisgender.

Adolescents assigned female at birth made up 80% of gender minority participants – and reported higher difficulties compared to those assigned male at birth within transgender and cisgender, but not nonbinary, identities.

**What's relevant?**

Health and education practitioners need to be aware that emotional and behavioural difficulties differ among gender minority identities, and that adolescents assigned female at birth are at an elevated risk.

## Supporting information


**Table S1.** Sample breakdown by gender identity (*n* = 122,766).
**Table S2.** Identities within ‘another gender identity’ category in sample before imputation.
**Table S3.** Associations between gender identity and emotional and behavioural difficulties (*n* = 122,766).
**Table S4.** Associations between gender identity and assigned sex at birth with emotional and behavioural difficulties (*n* = 122,766).
**Table S5.** Associations between gender identity and emotional and behavioural difficulties in the sample with no missing data.
**Table S6.** Associations between gender identity and assigned sex at birth with emotional and behavioural difficulties in the sample with no missing data.

## Data Availability

Data used in this study were obtained from The School Health Research Network (SHRN) at Cardiff University, UK. Data collected via The SHRN Student Health and Well‐being Survey are available for research purposes upon completion and approval of a data access request. Please contact shrn@cardiff.ac.uk for further information.
