# Supplementary material for: Emotional and behavioural difficulties in gender minority compared to cisgender adolescents: identity specific findings from a contemporary national study
Source: J Child Psychol Psychiatry. 2025 Sep 10;67(3):312–20. doi: 10.1111/jcpp.70050 (PMC12883586; doi:10.1111/jcpp.70050)
Supplement: Supplementary file 1 — Table S1. Sample breakdown by gender identity (n = 122,766). Table S2. Identities within ‘another gender identity’ category in sample before imputation. Table S3. Associations between gender identity and emotional and behavioural difficulties (n = 122,766). Table S4. Associations between gender identity and assigned sex at birth with emotional and behavioural difficulties (n = 122,766). Table S5. Associations between gender identity and emotional and behavioural difficulties in the sample with no missing data. Table S6. Associations between gender identity and assigned sex at birth with emotional and behavioural difficulties in the sample with no missing data. [file JCPP-67-312-s001.docx]

**Supplementary appendix**

1. Table S1. Sample breakdown by gender identity (n=122,766)
2. Table S2. Identities within ‘another gender identity’ category in sample before imputation.
3. Table S3. Associations between gender identity and emotional and behavioural difficulties (n=122,766)
4. Table S4. Associations between gender identity and assigned sex at birth with emotional and behavioural difficulties (n=122,766)
5. Table S5. Associations between gender identity and emotional and behavioural difficulties in the sample with no missing data
6. Table S6. Associations between gender identity and assigned sex at birth with emotional and behavioural difficulties in the sample with no missing data

**Table S1. Sample breakdown by gender identity (n=122,766)**

|  | **% (95% confidence interval)** | **n** |
| --- | --- | --- |
| *Cisgender* | *96.9* | *118,960* |
| Boy | 49.3 (49.3, 49.3) | 60,524 |
| Girl | 47.6 (47.5, 47.6) | 58,436 |
| *Gender minority* | *2.0* | *2,455* |
| Boy / transboy | 0.6 (0.6, 0.6) | 737 |
| Girl / transgirl | 0.3 (0.2, 0.3) | 367 |
| Non-binary AMAB | 0.1 (0.1, 0.1) | 123 |
| Non-binary AFAB | 0.6 (0.6, 0.6) | 737 |
| Another gender identity AFAB | 0.4 (0.4, 0.4) | 491 |
| *Unspecified gender* | *1.1* | *1,351* |
| Unspecified AMAB | 0.2 (0.2, 0.2) | 246 |
| Unspecified AFAB | 0.9 (0.9, 0.9) | 1105 |
| Total | 100.0 | 122,766 |

AMAB: assigned male at birth; AFAB: assigned female at birth

**Table S2. Identities within ‘another gender identity’ category in sample before imputation**

|  | **Another gender identity AMAB (n=43)** | **Another gender identity AFAB (n=447)** |
| --- | --- | --- |
| Agender | 8 | 37 |
| Bigender | 3 | 24 |
| Boyflux | 1 | 0 |
| Demi-boy | 8 | 23 |
| Demi-girl | 1 | 71 |
| Demigender | 0 | 3 |
| Femboy | 1 | 0 |
| Genderdoe | 0 | 1 |
| Genderfae | 0 | 2 |
| Genderfaun | 1 | 1 |
| Genderfluid | 18 | 250 |
| Genderflux | 0 | 3 |
| Gendergender | 0 | 1 |
| Genderqueer | 1 | 13 |
| Gendervoid | 0 | 1 |
| Intergender | 1 | 0 |
| Librafemme | 0 | 1 |
| Librafluid | 0 | 1 |
| Omnigender | 0 | 2 |
| Pangender | 0 | 5 |
| Paragirl | 0 | 2 |
| Polygender | 0 | 1 |
| Tomboy | 0 | 2 |
| Transmasculine | 0 | 3 |

**Table S3. Associations between gender identity and emotional and behavioural difficulties (n=122,766)**

|  |  | **Correlation coefficient (95% confidence interval)** | | | |
| --- | --- | --- | --- | --- | --- |
| **SDQ total** | **Cisgender** | **Transgender** | **Non-binary** | **Another gender identity** | **Unspecified gender** |
| M1a (unadjusted) | Ref | 5.18 (4.70, 5.65) | 7.91 (7.50, 8.32) | 8.08 (7.56, 8.60) | 5.73 (5.33, 6.12) |
| M1b (M1a + age) | Ref | 5.19 (4.73, 5.66) | 7.85 (7.44, 8.26) | 8.03 (7.51, 8.55) | 5.76 (5.36, 6.15) |
| M1c (M1a + ethnicity) | Ref | 5.20 (4.72, 5.67) | 7.91 (7.51, 8.32) | 8.08 (7.56, 8.60) | 5.74 (5.35, 6.14) |
| M1d (M1b + ethnicity & FAS) | Ref | 5.05 (4.58, 5.51) | 7.66 (7.25, 8.06) | 7.86 (7.34, 8.38) | 5.60 (5.21, 5.99) |

FAS: Family Affluence Scale. M: Model. Ref: Reference category.

**Table S4. Associations between gender identity and assigned sex at birth with emotional and behavioural difficulties (n=122,766)**

|  | **Correlation coefficient (95 % confidence interval)** | | | | | | | | |
| --- | --- | --- | --- | --- | --- | --- | --- | --- | --- |
|  | **Cisgender** | | **Gender minority** | | | | | **Unspecified gender** | |
| **SDQ total** | **Boy**  **(n=60,524)** | **Girl**  **(n=58,436)** | **Boy/ transboy**  **(n=737)** | **Girl/ transgirl**  **(n=367)** | **Non-binary**  **AMAB**  **(n=123)** | **Non-binary**  **AFAB**  **(n=737)** | **Another gender identity AFAB**  **(n=491)** | **Unspecified**  **AMAB**  **(n=246)** | **Unspecified AFAB**  **(n=1105)** |
| M1a  (unadjusted) | Ref | 2.32  (2.23, 2.42) | 7.26  (6.70, 7.82) | 4.01  (3.19, 4.83) | 7.86  (6.72, 8.99) | 9.27  (8.84, 9.71) | 9.22  (8.70, 9.74) | 5.05  (4.13, 5.97) | 7.33  (6.89, 7.77) |
| M1b  (M1a + age) | Ref | 2.32  (2.23, 2.42) | 7.28  (6.72, 7.83) | 4.02  (3.20, 4.85) | 7.82  (6.68, 8.96) | 9.22  (8.78, 9.65) | 9.17  (8.65, 9.69) | 4.90  (3.98, 5.82) | 7.41  (6.97, 7.85) |
| M1c  (M1a + ethnicity) | Ref | 2.32  (2.22, 2.41) | 7.27  (6.71, 7.82) | 4.05  (3.22, 4.87) | 7.87  (6.74, 9.00) | 9.27  (8.84, 9.70) | 9.21  (8.69, 9.74) | 5.12  (4.20, 6.03) | 7.33  (6.89, 7.77) |
| M1d  (M1b + ethnicity & FAS) | Ref | 2.30  (2.21, 2.39) | 7.08  (6.53, 7.63) | 3.98  (3.16, 4.80) | 7.61  (6.48, 8.74) | 9.01  (8.58, 9.44) | 8.99  (8.47, 9.51) | 4.84  (3.92, 5.76) | 7.21  (6.78, 7.66) |

AFAB: assigned female at birth; AMAB assigned male at birth; FAS: Family Affluence scale. M: Model; Ref: Reference category

**Table S5. Associations between gender identity and emotional and behavioural difficulties in the sample with no missing data**

|  |  |  | **Correlation coefficient (95% confidence interval)** | | | |
| --- | --- | --- | --- | --- | --- | --- |
| **SDQ total** | **n** | **Cisgender** | **Transgender** | **Non-binary** | **Another gender identity** | **Unspecified gender** |
| M1a (unadjusted) | 101,561 | Ref | 5.99 (5.51, 6.48) | 8.10 (7.70, 8.51) | 8.16 (7.64, 8.67) | 7.19 (6.83, 7.56) |
| M1b (M1a + age) | 100,104 | Ref | 5.95 (5.47, 6.43) | 8.06 (7.64, 8.47) | 8.10 (7.58, 8.62) | 7.22 (6.86, 7.59) |
| M1c (M1a + ethnicity) | 99,326 | Ref | 6.03 (5.53, 6.52) | 8.13 (7.72, 8.54) | 8.15 (7.63, 8.68) | 7.17 (6.80, 7.55) |
| M1d (M1b + ethnicity & FAS) | 93,769 | Ref | 5.74 (5.23, 6.25) | 7.93 (7.51, 8.35) | 7.84 (7.32, 8.37) | 6.98 (6.60, 7.35) |

FAS: Family Affluence Scale. M: Model; Ref: Reference category.

**Table S6. Associations between gender identity and assigned sex at birth with emotional and behavioural difficulties in the sample with no missing data**

|  |  |  | | **Correlation coefficient (95 % confidence interval)** | | | | |  | |
| --- | --- | --- | --- | --- | --- | --- | --- | --- | --- | --- |
|  |  | **Cisgender** | | **Gender minority** | | | | | **Unspecified gender** | |
| **SDQ total** | **n** | **Boy** | **Girl** | **Boy/ transboy** | **Girl/ transgirl** | **Non-binary AMAB** | **Non-binary**  **AFAB** | **Another gender identity AFAB** | **Unspecified AMAB** | **Unspecified AFAB** |
| M1a (unadjusted) | 101,561 | Ref | 2.70  (2.60, 2.80) | 8.23  (7.65, 8.81) | 4.86  (3.96, 5.75) | 8.39  (7.30, 9.47) | 9.63  (9.20, 10.07) | 9.50  (8.99, 10.01) | 6.94  (5.94, 7.94) | 8.85  (8.44, 9.26) |
| M1b (M1a + age) | 100,104 | Ref | 2.71  (2.61, 2.81) | 8.21  (7.63, 8.79) | 4.82  (3.92, 5.72) | 8.38  (7.30, 9.46) | 9.58  (9.14, 10.03) | 9.45  (8.93, 9.97) | 6.72  (5.71, 7.73) | 8.94  (8.53, 9.35) |
| M1c (M1a + ethnicity) | 99,326 | Ref | 2.72  (2.61, 2.82) | 8.25  (7.66, 8.84) | 4.97  (4.06, 5.88) | 8.48  (7.38, 9.59) | 9.66  (9.22, 10.10) | 9.50  (8.98, 10.03) | 6.99  (5.97, 8.02) | 8.82  (8.41, 9.24) |
| M1d (M1b + ethnicity & FAS) | 93,769 | Ref | 2.73  (2.62, 2.83) | 7.95  (7.35, 8.56) | 4.76  (3.80, 5.71) | 8.66  (7.57, 9.74) | 9.41  (8.95, 9.86) | 9.21  (8.68, 9.73) | 6.70  (5.66, 7.74) | 8.66  (8.22, 9.09) |

Note: AFAB: assigned female at birth; AMAB assigned male at birth; FAS: Family Affluence Scale. M: Model; Ref: Reference category
